# Influence of an acute fast on ambulatory blood pressure and autonomic cardiovascular control

**DOI:** 10.1152/ajpregu.00283.2021

**Published:** 2022-04-05

**Authors:** Joshua Eric Gonzalez, William Harold Cooke

**Affiliations:** ^1^Oregon Institute of Occupational Health Sciences, Oregon Health & Science University, Portland, Oregon; ^2^Department of Kinesiology & Integrative Physiology, Michigan Technological University, Houghton, Michigan; ^3^Health Research Institute, Michigan Technological University, Houghton, Michigan

**Keywords:** autonomic control, fasting, food deprivation, muscle sympathetic nerve activity

## Abstract

Evidence suggests that intermittent fasting improves cardiovascular health by reducing arterial blood pressure, but contributing mechanisms are unclear. The purpose of this study was to determine the influence of an acute fast on hemodynamics, muscle sympathetic nerve activity (MSNA), and autonomic control at rest and during an arterial pressure challenge. Twenty-five young normotensive volunteers were tested twice, in the fed and fasted (24 h) states (randomized). Twenty-four hour ambulatory blood pressure was measured before an autonomic function test, which consisted of a 10-min period of controlled breathing (CB) at 0.25 Hz followed by 3, 15-s Valsalva maneuvers (VMs). We recorded the ECG, beat-to-beat arterial pressure, and MSNA throughout the autonomic test. Vagal-cardiac modulation via heart rate variability (HRV) was assessed in both time and frequency domains, cardiovagal baroreflex sensitivity (cvBRS) was assessed with linear regression, and stroke volume was estimated from pulse contour. All fed versus fasted comparisons presented are different at *P* < 0.05. Fasting reduced ambulatory mean arterial pressure (81 ± 1 vs. 78 ± 1 mmHg) and heart rate (69 ± 2 vs. 65 ± 2 beats/min). CB revealed enhanced HRV through increased R-R intervals (992 ± 30 vs. 1,059 ± 37 ms) and normalized high frequency (HFnu) R-R interval spectral power (55 ± 3 vs. 62 ± 3%). Estimated stroke volume was higher after fasting (by 13%) as was cvBRS (20 ± 2 vs. 26 ± 5 ms/mmHg) and cvBRS during phase IV of the VM (9 ± 1 vs. 12 ± 1 ms/mmHg). MSNA (*n* = 12) did not change (16 ± 11 vs. 15 ± 8 bursts/min; *P* = 0.18). Our results show that acute fasting is consistent with improved cardiovascular health: such improvements are driven by reduced ambulatory arterial pressure and enhanced vagal-cardiac modulation.

## INTRODUCTION

Obesity is a multifaceted disorder that is a risk factor for the development of cardiovascular disease and is characterized by sympathetic overactivity (1, 2). Recently, intermittent fasting has gained popularity as a weight reduction strategy in the United States. A 2020 survey by the International Food Informational Council reported that intermittent fasting is the most common dieting strategy among Americans, surpassing clean eating and the ketogenic diet (3). Fasting is defined as a period of complete caloric cessation, commonly for 12 h or longer, with ad libitum access to water (4). Intermittent fasting promotes weight loss and increases fat oxidation in individuals with obesity and nonobese individuals (5, 6). However, the health benefits of intermittent fasting may go beyond weight loss. Recently, multiple reviews have highlighted the cardiovascular benefits of chronic intermittent fasting (7–9). In humans, intermittent fasting can reduce blood pressure, oxidative stress, and the risk of atherosclerosis (5, 10–12). One month of alternate-day fasting effectively lowers blood pressure and heart rate in healthy nonobese humans, suggesting that chronic fasting may enhance parasympathetic activity (13). Although evidence supports the benefits of chronic intermittent fasting on cardiovascular health, few studies have investigated the influence of a single bout of acute fasting. The autonomic nervous system (ANS) plays a key role in the regulation of arterial pressure and is one of the primary contributors to cardiovascular homeostasis. However, the influence of fasting on the ANS and the mechanisms responsible for the observed reductions in blood pressure are unclear.

In humans, the influence of fasting on autonomic balance has not been fully elucidated. When compared with food consumption, an overnight fast (∼12 h) enhances parasympathetic modulation of the heart as suggested by spectral analysis of heart rate variability (HRV; 14, 15). Only one previous study has directly measured sympathetic neural outflow via microneurography in fasted humans. In 11 obese hypertensive women, a 48-h fast increased muscle sympathetic nerve activity (MSNA) moderately, and reduced blood pressure (16). It is, therefore, necessary to reevaluate MSNA-blood pressure associations in normal weight, normotensive individuals, and we also observe that the influence of an acute fast on cardiovagal baroreflex sensitivity (cvBRS) is unknown. It is clear that primary associations between autonomic control mechanisms and ambulatory blood pressure responses to an acute fast have not yet been described. Elucidating the influence of a single bout of acute fasting on prognostic indicators of cardiovascular health could provide mechanistic insight into how chronic intermittent fasting improves health.

Accordingly, the primary aim of this study was to determine the influence of an acute 24-h fast on ambulatory blood pressure and autonomic cardiovascular and neurovascular control. We assessed HRV, cardiovagal baroreflex sensitivity (cvBRS), and MSNA to test the hypothesis that ambulatory blood pressure reductions after an acute 24-h fast are associated with increased cvBRS and HRV, and reduced MSNA.

## METHODS

### Participants

Twenty-five healthy young adults (14 M and 11 F, age of 23 ± 3 yr, height of 176 ± 16 cm, weight of 76 ± 16 kg, body mass index (BMI) of 24 ± 4 kg/m^2^, and body surface area (BSA) of 1.9 ± 0.2 m^2^) participated. All participants had no history of autonomic dysfunction, hypertension, respiratory disease, diabetes, tobacco, or vaporized nicotine usage and were not taking any prescription medications. Participants were screened for hypertension (systolic <130 and/or diastolic < 80) via three seated blood pressures taken with an automated sphygmomanometer (Omron Healthcare, Lake Forest, IL). All female participants were tested during the early follicular phase of their menstrual cycle. Participants were instructed to abstain from exercise and caffeine for 24 h before laboratory testing. All participants signed a consent form that had been approved by the Institutional Review Board for human subject research at Michigan Technological University.

### Measurements

#### Ambulatory blood pressure monitoring and actigraphy.

Twenty-four-hour ambulatory blood pressure monitoring (ABPM; 90217A Ambulatory Blood Pressure Monitor, Spacelabs Healthcare, Snoqualmie, WA) and actigraphy data (Actiwatch Spectrum Pro, Phillips Respironics, Bend, OR) were collected twice leading up to the autonomic test. ABPM and actigraphy data were collected when the participant ate their normal diet for 24 h (ABPM-fed), and then again when the participant was fasting for 24 h (ABPM-fasted): data collection periods were separated by 4 wk. Sampling frequency was set to collect brachial blood pressures every 20 min between 0700 and 2200 h and every 30 min from 2200 to 0700 h. Participants were instructed to immobilize their arm upon cuff inflation and to only remove the cuff and the actiwatch during showering. Wake and sleep blood pressure values were defined and adjusted to the sleep and wake times recorded by the actiwatch. A minimum of 20 valid wake recordings and seven valid night recordings during both conditions was required for a satisfactory ABPM recording (17).

#### Blood Biomarkers and Hydration Status.

Upon arrival at the laboratory for autonomic testing, blood glucose, ketones, and lipids were measured. Whole blood was utilized to measure total cholesterol, high-density lipoprotein, low-density lipoproteins, triglycerides, and glucose using a Cholestech LDX analyzer (Alere Cholestech, San Diego, CA). Whole blood was also utilized to measure blood ketones (β-OHB) using a Precision Xtra (Abbott Laboratories, MediSense Products, Inc., Bedford, MA) ketone monitor. Urine was also collected to assess hydration status via urine specific gravity, using a PALS-10S urine refractometer (Atago, Tokyo, Japan).

#### Blood Pressure and Heart Rate.

Beat-to-beat arterial blood pressure was recorded continuously throughout all time points of the autonomic function test using a NOVA Finometer (Finapres Medical Systems, Amsterdam, The Netherlands). Heart rate was recorded via a three-lead electrocardiogram, and respiratory rate was continuously measured using a pneumobelt. Three brachial blood pressures (HEM-907XL, Omron, Kyoto, Japan) were used to calibrate finger plethysmography. Data were sampled at 500 Hz (WINDAQ, Dataq Instruments, Akron, OH) and analyzed with specialized software (WINCPRS, Absolute Aliens, Turku, Finland). R waves generated from the ECG signal were automatically detected and marked. Heart rate variability was assessed in the frequency domain using a Fourier transform with a Hann window. Heart rate variability was quantified by calculating the total integrated area under the R-R interval power spectrum from 0.04 to 0.4 Hz. Integrated areas were separated into high-frequency (HF, 0.15–0.4 Hz) and low-frequency (LF, 0.04–0.15 Hz) bands. Systolic arterial pressure at the low frequency (SAPLF, 0.04–0.15 Hz) was calculated as an additional estimate of peripheral sympathetic activity (18). To compare R-R interval power spectra more accurately between participants whose total power may vary widely, we normalized our data by dividing integrated LF and HF spectra by the total power and multiplying by 100. Systolic and diastolic arterial pressures were marked from the Finometer tracings. With the use of the arterial pressure waveform as an input, stroke volume, cardiac output, and total peripheral resistance were automatically estimated on a beat-to-beat basis using the pulse contour method (19).

#### Microneurography.

Multifiber efferent sympathetic nerve traffic was recorded from the peroneal nerve muscle fascicles at the popliteal fossa by inserting a tungsten microelectrode (Frederick Haer and Co., Bowdoinham, ME). A reference electrode was inserted subcutaneously 2–3 cm from the recording electrode. Both electrodes were connected to a differential preamplifier and then to an amplifier (total gain 80,000) where the nerve signal was band-pass filtered (700–2,000 Hz) and integrated (time constant, 0.1 s) to obtain a mean voltage display of nerve activity. Satisfactory recordings of MSNA were defined by spontaneous pulse-synchronous bursts that did not change during tactile or auditory stimulation and increased during end-expiratory apnea. Muscle sympathetic nerve bursts were automatically detected based on their amplitude, and a 1.3 s expected burst peak latency from the previous R wave. All automated detection results were checked manually. Sympathetic bursts of activity were expressed as burst frequency (bursts/min) and burst incidence (bursts/100 heartbeats).

### Experimental Design

This study was a randomized controlled crossover design with repeated measures. Each participant was tested twice, once in the fed condition (3-h postprandial) and once in the fasted condition (24-h postprandial). Trial order (fed vs. fasted) was randomized, and participants were informed 3 days before scheduled autonomic testing to which condition they would be assigned. Tests were performed ∼4 wk apart. Participants reported to the laboratory 24 h before their scheduled autonomic test to be fitted with an actigraphy watch and ambulatory blood pressure cuff that they wore for 24 h leading up to the autonomic test. A standardized caloric-controlled meal was provided for each condition. The caloric intake of the standardized meal was estimated to be one-third of the total caloric intake needed to maintain the individual participants’ weight. Macronutrient composition of the meal was estimated to be ∼55% carbohydrate, ∼20% fats, and ∼25% protein. To estimate total daily energy needs, resting metabolic rate was estimated using the Mifflin-St. Jeor equation and multiplied by a physical activity factor (20, 21). Participants were instructed to intake water ad libitum.

**Figure 1.**
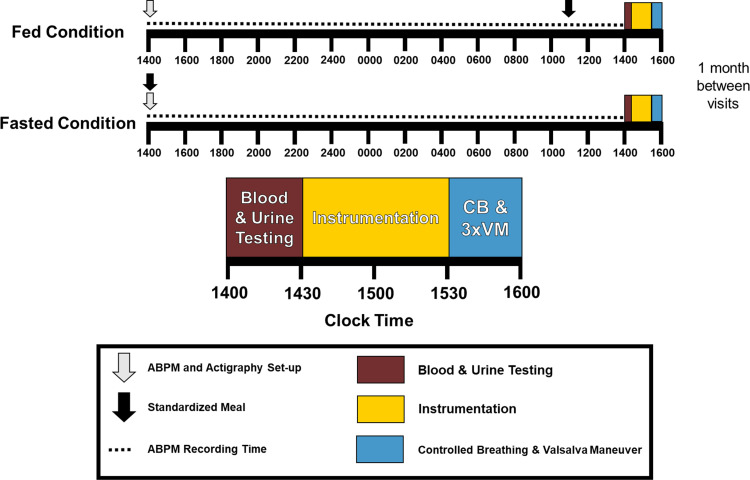
Experimental design of the present study including 24-h ambulatory blood pressure monitoring leading up to an autonomic function test consisting of 10 min of controlled breathing and three Valsalva maneuvers. Timing for the fed and fasted condition are represented. Experimental conditions were randomized and separated by ∼4 wk. ABPM, ambulatory blood pressure monitoring; CB, controlled breathing; VM, Valsalva maneuvers.

Upon arrival at the laboratory, participants turned in 24-h measurement equipment, and blood and urine samples were collected and analyzed. Participants were then situated in a supine position on a cushioned laboratory table for hemodynamic and microneurographic instrumentation. After instrumentation, participants were provided a minimum of 5-min unrecorded rest to confirm hemodynamic and neural stability. Three supine brachial blood pressures (HEM-907XL, Omron, Japan) were used to calibrate finger plethysmography. During the controlled breathing (CB) portion of the experiment, participants breathed in time to a computer display showing a waveform set at 0.25 Hz (15 breaths/min). Controlled breathing was followed by three Valsalva maneuvers (VMs) each separated by 1 min of rest. The experimental protocol is summarized in Fig. 1.

### Valsalva Maneuver

**Figure 2.**
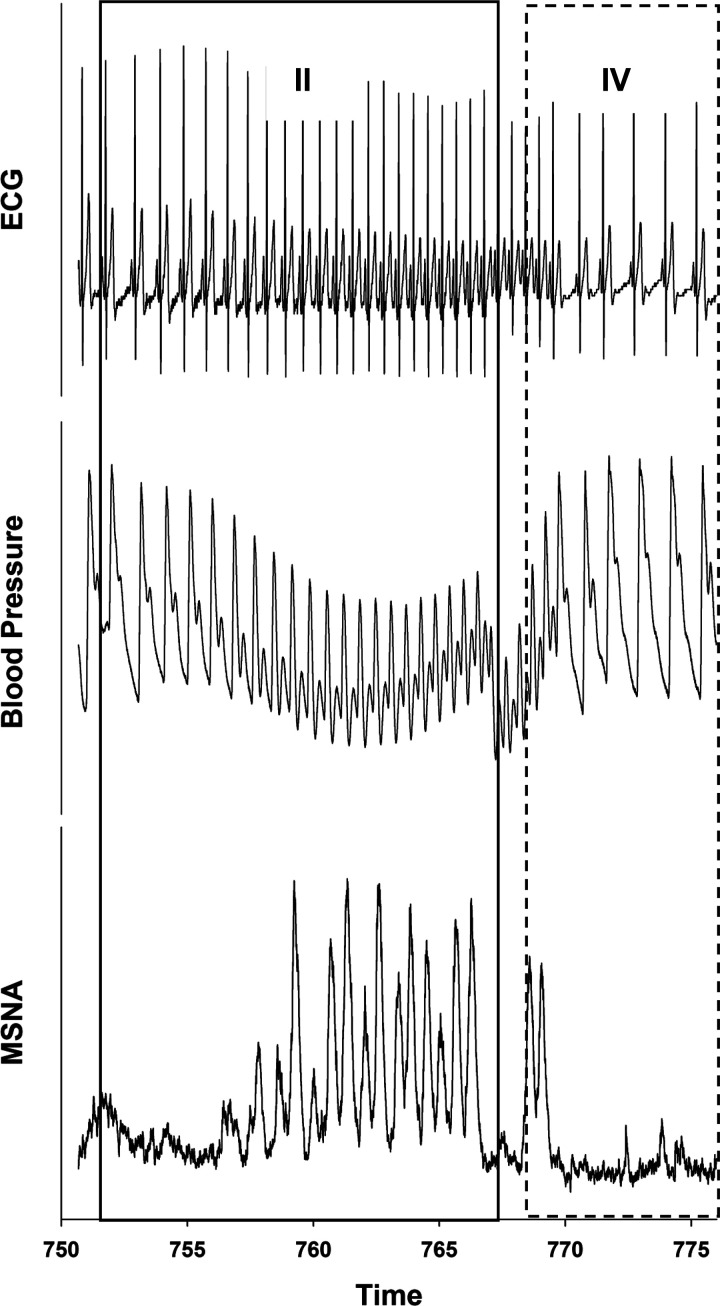
Representative ECG, blood pressure (BP), and muscle sympathetic nerve activity during the 15-s Valsalva maneuver (VM). Phase II of the VM is delineated by a solid line, which contains the highest BP value upon strain initiation and the first BP decrease upon strain termination. Phase IV of the VM is delineated by a dotted line, which contains the first BP increase after strain termination and the first BP decrease after the phase IV overshoot. MSNA, muscle sympathetic nerve activity.

During each VM, participants were asked to forcefully exhale to 40 mmHg for 15 s into a mouthpiece attached to an analog manometer. A nose clip was used to seal the nose, and a small leak was allowed in the manometer to keep the glottis open during each strain. Each of the three Valsalva maneuvers was separated by a 1-min recovery of spontaneous breathing. For each participant, we identified the four Valsalva phases as outlined by Smith et al. (22). Phase II began at the highest systolic pressure value recorded at the onset of strain and ended with abrupt pressure reductions at the end of the 15-s strain period. Phase IV began at the first systolic value above the last value recorded during phase II and ended at the first decrease in systolic pressure following overshoot. We evaluated cardiovagal baroreflex sensitivity during phase II and phase IV using the slope method described by Kautzner (23). Briefly, cardiovagal baroreflex sensitivity during each VM was calculated using the linear relationship between R-R interval and systolic arterial pressure (SAP) for both phase II (hypotensive stimulus) and phase IV (hypertensive stimulus). To be considered a valid sequence, correlation coefficients were set at >0.70, and only VM that demonstrated morphological and temporal consistency were included in the analysis. In addition, a minimum reduction of 15 mmHg during phase II was required for inclusion (Fig. 2).

### Spontaneous Cardiovagal Baroreflex Sensitivity

Spontaneous cardiovagal BRS was assessed via the sequence method (24) during the 10-min of controlled breathing. Within the time domain, the baroreflex sensitivity was assessed by identifying sequences of three or more consecutively increasing systolic arterial pressures (SAPs) to three or more consecutive-lengthening R-R intervals (up-up sequences and vagal activation). In addition, systolic pressures that exhibited three or more decreases in SAP and three or more shortenings of R-R interval were identified (down-down sequences and vagal inhibition). SAP that changed by at least 1 mmHg/beat and R-R interval that changed by at least 4 ms were identified as a sequence. A linear regression analysis of each sequence of R-R interval and SAP determined the slope. A minimum *r* value of >0.7 was used as criteria for accepting a sequence.

### Statistical Analysis

Using a two-sample *t* test to calculate power based on published literature (15), we estimated that a sample size of 23 subjects would give us sufficient power (α = 0.05, β = 0.95) to test our hypothesis. Statistical analyses were performed with Sigmaplot 14.0 (Systat Software, San Jose, CA) and Graphpad Prism (Graphpad, Software, Inc., San Diego, CA). Normality was assessed using a Shapiro–Wilk test. If data were found to be normally distributed, dependent variables were assessed with a paired *t* test. If data were not normally distributed a Wilcoxon matched-pairs signed-rank test was used to assess dependent variables. Blood biomarkers used to assess fasting compliance were analyzed via two-tailed statistical tests. One-tailed statistical tests were utilized for directional hypotheses related to reduced blood pressure, MSNA, heart rate, increased HRV and cvBRS. *P* values <0.05 were considered statistically significant for all tests.

## RESULTS

### Blood Biomarkers and Hydration

Participants were weight stable and similarly hydrated between conditions. Urine specific gravity was collected in 23 participants (two participants could not produce a urine sample at the start of experimentation). To assure fasting compliance, glucose, blood ketones, and lipids were measured. Blood triglycerides and glucose were significantly decreased, and ketones were increased in the fasted condition compared with the fed condition. Participants’ characteristics and blood biomarkers from the fed and fasted conditions are displayed in Table 1.

**Table 1.** Participant blood biomarkers in the fed and fasted condition

Variable	Fed	Fasted	*t* Test *P* Value	Wilcoxon *P* Value
Weight, kg	76.3 ± 15.4	75.6 ± 15.2	0.271	
Urine specific gravity, AU (*n* = 23)	1.015 ± 0.002	1.011 ± 0.002	0.113	
Total cholesterol, mg/dL	172.4 ± 35.2	172.7 ± 29.1	0.937	
Low-density lipoprotein, mg/dL	95.6 ± 29.9	105.5 ± 21.5	0.253	
High-density lipoprotein, mg/dL	58.8 ± 18.9	56.2 ± 20.3	0.151	
Triglycerides, mg/dL	120.8 ± 61.6	77.2 ± 37.9		0.003
Glucose, mg/dL	98.8 ± 11.7	80.9 ± 8.0	<0.001	
Ketones, mmol/L (β-hydroxybutyrate)	0.128 ± 0.13	0.536 ± 0.49		<0.001

Values are means ± SD. *n* = 25, unless specified in table. Paired *t* tests were used to compare variables between the fed and fasted condition. Wilcoxon matched-pairs signed rank test was used to compare variables not normally distributed. Two-tailed *P* values are displayed. AU, arbitrary units.

### Ambulatory Blood Pressure

**Figure 3.**
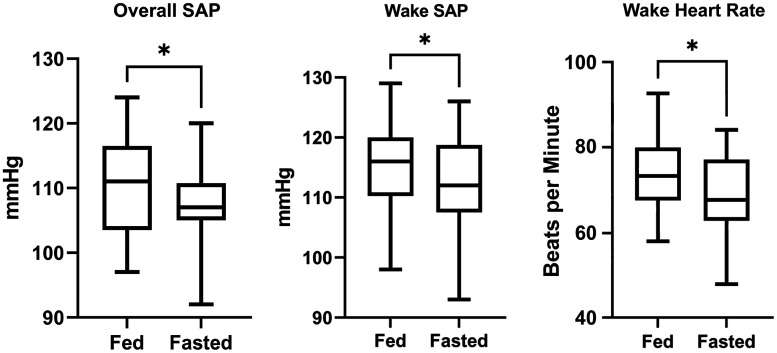
Overall systolic blood pressure, wake systolic blood pressure, and wake heart rate represented as box plots. The line in the boxplots represents the median, and the box represents the interquartile range (IQR: the difference between the 25th and 75th percentile). The whiskers extend from the upper and lower edge of the box to the highest and lowest values. *n* = 20 human subjects. SAP, systolic arterial pressure. *P < 0.05.

For inclusion in the data set, we required a minimum of 20 wake measurements and seven sleep measurements. The average number of wake recordings was 32 ± 7 for the ABPM-fed condition and 34 ± 8 for the ABPM-fasted condition (Table 2). The average number of sleep recordings was 13 ± 3 for the ABPM-fed condition and 15 ± 5 for the ABPM-fasted condition with a 74 ± 12% reading success rate for the ABPM-fed condition and a 78 ± 13% success rate for the ABPM-fasted condition. Twenty participants were included in this data set (two were eliminated for device failure and three for not meeting minimum requirements). Actigraphy was utilized to calibrate the ABPM data points to participant wake and sleep times. In young healthy normotensive individuals, fasting reduced overall ambulatory systolic (−2 ± 1 mmHg; *P* = 0.02), diastolic (−2 mmHg; *P* = 0.04) and mean arterial pressure (−2 ± 1 mmHg; *P* = 0.02) compared with the ABPM-fed condition. Heart rate, measured from the ambulatory cuff, was also decreased in the fasted condition. These reductions in overall blood pressure were primarily driven by reductions in wake SAP (seen in Fig. 3). Fasting did not affect blood pressure during sleep or blood pressure dipping patterns.

**Table 2.** Overall 24-h ambulatory blood pressure in the fed and fasted condition

Variable	ABPM-Fed	ABPM-Fasted	*P* Value
Overall SAP, mmHg	110 ± 8	108 ± 7	0.023
Overall DAP, mmHg	64 ± 6	63 ± 6	0.037
Overall MAP, mmHg	81 ± 5	78 ± 5	0.020
Overall pulse pressure, mmHg	47 ± 6	46 ± 6	0.053
Overall heart rate, beats/min	69 ± 8	65 ± 10	0.001

Values are means ± SD. *n* = 20. Paired *t* tests were used to compare variables between the fed and fasted condition. One-tailed *P* values are displayed. ABPM, ambulatory blood pressure monitoring; DAP, diastolic arterial pressure; MAP mean arterial pressure; SAP, systolic arterial pressure.

### Controlled Breathing and the Valsalva Maneuver

**Figure 4.**
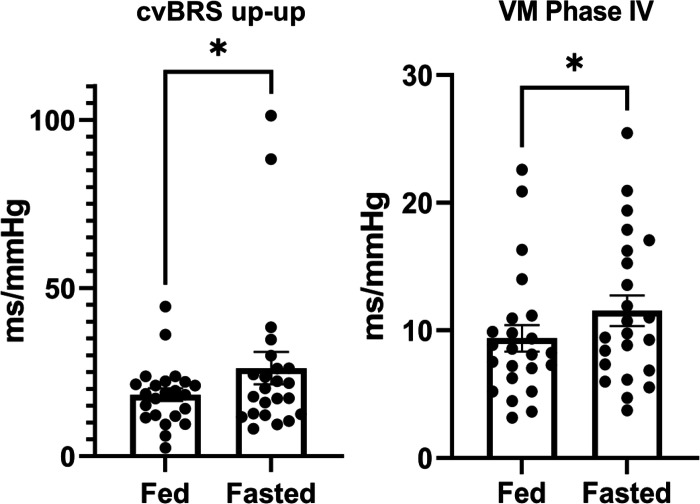
Cardiovagal baroreflex sensitivity up-up (cvBRS up-up, *n* = 23) represented as individual data points and bar graphs (means ± SE). cvBRS up-up was not normally distributed, thus a Wilcoxon signed ranked test was used for comparison. Valsalva maneuver phase IV (VM phase IV, *n* = 22) SAP-RRI slope represented as individual data points and bar graphs (means ± SE). SAP, systolic arterial pressure. *P < 0.05.

During controlled breathing in the fasted condition, R-R interval and normalized HF R-R interval spectral power measured via frequency domain analysis were increased. Absolute HF power was not different. Absolute HF and normalized HF power are shown for comparison in Table 3. In the fasted condition, normalized high frequency (HFnu) significantly increased, and normalized low frequency (LFnu) decreased. Blood pressure and MSNA (*n* = 12) were similar during controlled breathing between conditions, but SAPLF power decreased in the fasted condition. Stroke volume estimated from the pulse contour method was increased in the fasted condition. The increase in stroke volume did not significantly alter estimated cardiac output or total peripheral resistance between conditions (seen in Table 3). In addition, spontaneous cardiovagal baroreflex sensitivity calculated from SAP-RRI up sequences was significantly increased in the fasted condition (seen in Fig. 4). cvBRS down sequences exhibited a strong trend to increase in the fasted condition but did not meet the criteria for statistical significance (*P* = 0.07).

**Table 3.** Average autonomic cardiovascular and hemodynamic data recorded during controlled breathing in the fed and fasted condition

Variable	Fed	Fasted	*t* Test *P* Value	Wilcoxon *P* Value
R-R interval, ms	992 ± 151	1,059 ± 186	0.024	
SAP, mmHg	107 ± 12	107 ± 11	0.481	
SAPLF, mmHg^2^/Hz	4.3 ± 3.4	2.5 ± 2		0.03
DAP, mmHg	57 ± 9	58 ± 10	0.431	
MSNA burst frequency, beats/min, (*n* = 12)	16 ± 11	15 ± 8	0.180	
MSNA burst incidence, b/100 heartbeats, (*n* = 12)	23 ± 16	26 ± 14	0.426	
RRIHFnu, %	55 ± 13	62 ± 16	0.022	
RRILFnu, %	46 ± 13	38 ± 16	0.022	
RRI HF, ms^2^/Hz	1,832 ± 1,523	2,717 ± 4,779		0.13
Stroke volume, mL	88 ± 20	100 ± 26		0.011
Cardiac output, L/min	5.3 ± 1.3	5.7 ± 1.4	0.084	
Total peripheral resistance, mmHg·min/L	15 ± 4	14.5 ± 4	0.309	
cvBRS up-up, ms/mmHg, (*n* = 23)	20 ± 12	26 ± 23		0.029
cvBRS down-down, ms/mmHg	17 ± 8	21 ± 12		0.076

Values are means ± SD. *n* = 25, unless specified in table. Paired *t* tests were used to compare variables between the fed and fasted condition. Wilcoxon matched-pairs signed rank was test used to compare variables not normally distributed. One-tailed *P* values are displayed. cvBRS up-up and cvBRS down-down, cardiovagal baroreflex sensitivity up and down sequences; MSNA, muscle sympathetic nerve activity; RRIHF, absolute R-R interval oscillations at the high frequency; RRIHFnu and RRILFnu, R-R interval oscillations at the high and low frequency, normalized to total power; SAP, systolic arterial pressure; SAPLF, systolic arterial pressure oscillations at the low frequency.

Twenty-two participants were included in the Valsalva analysis. Three participants were excluded because they did not have SAP-RRI correlations above 0.70. Average SAP-RRI correlation coefficients for included participants were 0.88 for phase II and 0.81 for phase IV. Phase II MSNA burst frequency, ΔSAP, ΔRRI, and cardiovagal baroreflex sensitivity were similar between conditions. Phase IV ΔSAP was similar between conditions, however, ΔRRI was increased in the fasted state. In addition, enhanced cardiovagal baroreflex sensitivity was observed during phase IV of the Valsalva maneuver (Seen in Fig. 4 and Table 4), or the hypertensive component.

**Table 4.** Average cardiovagal baroreflex sensitivity during phase II and IV of the Valsalva maneuvers in the fed and fasted condition

Variable	Fed	Fasted	*t* Test *P* Value	Wilcoxon *P* Value
Phase II ΔRRI, ms	387 ± 118	389 ± 123	0.456	
Phase II ΔSAP, mmHg	31 ± 11	36 ± 20	0.131	
Phase II MSNA, bursts/min, (*n* = 12)	20 ± 8	18 ± 5	0.239	
Phase II cvBRS, ms/mmHg	11 ± 4	12 ± 7		0.474
Phase IV ΔRRI, ms	414 ± 202	484 ± 195	0.026	
Phase IV ΔSAP, mmHg	37 ± 14	42 ± 21	0.113	
Phase IV cvBRS, ms/mmHg	9 ± 5	12 ± 6	0.010	

Values are means ± SE. *n* = 22, unless specified in table. Paired *t* tests were used to compare variables between the fed and fasted condition. Wilcoxon matched-pairs signed rank was test used to compare variables not normally distributed. One-tailed *P* values are displayed. cvBRS, cardiovagal baroreflex sensitivity; MSNA, muscle sympathetic nerve activity; SAP, systolic arterial pressure.

## DISCUSSION

We tested young, healthy, normotensive individuals twice, once in a fasted condition (24-h postprandial) and once in a fed condition (3-h postprandial). Our purpose was to determine how acute fasting influences ambulatory blood pressure and autonomic cardiovascular and neurovascular control. We report four novel findings: First, fasting reduces overall ambulatory SAP, DAP, MAP, and HR. The measured reductions in overall 24-h blood pressure are driven primarily by reductions in wake SAP and HR. Second, acute fasting increases vagal modulation at the heart without altering directly measured peripheral sympathetic nerve activity, although estimates derived from SAPLF provide indirect evidence of peripheral sympathetic reductions. Specifically, fasting is associated with increased RRI and HFnu with no change in MSNA. Third, fasting enhances cvBRS measured both spontaneously during controlled breathing (cvBRS-up) and when baroreceptors are challenged with a naturally induced hypertensive stimulus (VM phase IV and cvBRS). Fourth, estimated stroke volume is increased in the fasted condition with no changes in blood pressure, cardiac output, or peripheral resistance during controlled breathing.

Chronic intermittent fasting regimens of 8–12 wk have been reported to reduce blood pressure (8). Our study is the first to report an acute reduction in blood pressure over a 24-h fasting period. The reductions in overall 24-h arterial pressure measured via ABPM in our normotensive sample population are mild (−2 mmHg SAP, −2 mmHg DAP, and −2 mmHg MAP) but are similar in magnitude to postexercise hypotension measured after a single bout of resistance exercise (−2 mmHg SAP; 25). Individuals seeking to lower their blood pressure especially struggle to reduce SAP (26). Thus, it is important to recognize that acute fasting primarily reduces the systolic component of blood pressure. Although the clinical significance of the mild reduction in 24-h blood pressure during fasting is uncertain, studies have reported that even small reductions in blood pressure (−1 mmHg SAP or −2 mmHg DAP) decrease the risk of cardiovascular disease (27, 28). Indeed, an epidemiological study and separate meta-analysis reported that periodic fasting (1 day/mo) is associated with a lower prevalence of coronary artery disease (29, 30). In addition, individuals who engage in intermittent fasting could benefit from the transient, but regular weekly time periods of reduced blood pressure. Recently, a study demonstrated that medically supervised fasting lasting 4 to 41 days (average 10 days) can reduce blood pressure in normotensive individuals to a degree similar to what we measured over the 24-h fast (−3 mmHg SAP and −2 mmHg DAP; 31). In hypertensive individuals, prolonged fasting dramatically reduces blood pressure, indicating that the benefits may scale with the severity of hypertension (31).

In normotensive rats, the hypotensive effect of fasting is primarily driven by suppression of cardiac sympathetic activity (32). The suppression of cardiac sympathetic activity (measured via norepinephrine turnover) decreases energy utilization and conserves calories (33). In humans, studies have reported conflicting evidence regarding the influence of fasting on sympathovagal balance at the level of the heart. Herbert et al. (34) reported a decrease in HFnu after 24 h of fasting and Mazurak et al. (35) reported a trend to increase HRV metrics after 24 h of fasting. However, both studies reported results from females only and only reported HRV metrics without cardiovagal baroreflex measures. Our results show that fasting enhances vagal modulation of the heart and improves spontaneous and dynamic cardiovagal baroreflex sensitivity. Enhanced cvBRS could act to buffer blood pressure perturbations throughout the day, which could lower overall average blood pressure during the fast. Previous research has demonstrated that there is a significant inverse relationship between cvBRS and ambulatory MAP in young healthy normotensive adults (36). We, therefore, propose that enhanced reflexive vagal activation at the heart to perturbations in blood pressure provides a mechanism for the observed reductions in overall ambulatory blood pressure measured while participants were fasting.

The Valsalva maneuver is an easily administered and reliable hemodynamic stressor that provokes both hypotensive (phase II) and hypertensive (phase IV) stimuli (22). The Valsalva maneuver reflects daily life activities such as straining, lifting, sneezing, or coughing in a reproducible experimental manner. In support of our measured increase in cvBRS-up during controlled breathing, we also report enhanced cvBRS during the phase IV overshoot of the VM. The phase IV overshoot of the VM activates the baroreceptors and induces vagally mediated bradycardia to buffer blood pressure. Crucially, the magnitude of the Valsalva stimulus on phase II MSNA and phase II and IV SAP were similar between conditions, indicating that vagal adaptation is responsible for the enhanced phase IV BRS and phase IV ΔRRI. The concomitant enhanced gain in cvBRS during both controlled breathing and the Valsalva maneuver suggests that fasting acutely improves cardiac autonomic control of blood pressure.

Contrary to our hypothesis, we did not observe a change in directly measured peripheral sympathetic outflow at rest between the fed and fasted conditions. We did, however, find that SAPLF is reduced with fasting. Low-frequency systolic pressure oscillations are linked directly to peripheral sympathetic activity in dogs (37) and with MSNA variability in humans (18). However, although low-frequency systolic pressure oscillations are linked to MSNA in humans studied as a group, taken individually the relationship between low-frequency systolic pressure oscillations and MSNA is not at all clear (38). Therefore, assumptions that the reductions we document in SAPLF are associated with reduced peripheral sympathetic activity in the current study should be interpreted with caution. To date, only one study has examined resting MSNA in fed and fasted individuals (16). Andersson et al. (16) reported that 48 h of fasting decreased blood pressure and increased MSNA slightly, with MSNA being measured in six moderately obese hypertensive women. During controlled breathing, we did not document any differences in MSNA or blood pressure between the fasted and fed conditions. In animals, fasting can induce a reciprocal sympathetic outflow, suppressing cardiac sympathetic activity, and enhancing secretion of epinephrine and norepinephrine from the adrenal medulla (39). The suppression in cardiac sympathetic activity conserves calories and the increase in circulating catecholamines, in conjunction with a fall in insulin, promotes substrate mobilization for energy production (40). Such disparate sympathetic activation at different target organs in response to fasting in humans is also possible. Therefore, it is reasonable to speculate that in the fasted state, parasympathetically mediated cardiovascular control is enhanced, and peripheral sympathetic outflow is unaffected and/or disassociated. In addition, it is possible that 24 h of food deprivation is not a sufficient stimulus to initiate sympathetic activation centrally or in the periphery. There is evidence in humans to support the notion that as fasting time exceeds 24 h, a shift toward sympathetic activation may occur at the heart and in the periphery. In participants who fasted for 48 h, Mazurak et al. (35) reported decreased HRV (measured via SDNN and RMSSD), and Andersson et al. (16) reported increased MSNA. A study by Webber and Macdonald (41) found that as fasting time approached 72 h, heart rate, epinephrine, and norepinephrine significantly increased compared with an overnight 12-h fast. In addition, multiple studies have demonstrated that metabolic rate increases at 36–48 h fast (41, 42). The shift toward sympathetic activation and increased metabolic rate as fasting time prolongs is likely caused by the heightened metabolic cost of gluconeogenesis and ketogenesis.

In the fasted state, ketone bodies are utilized as the main energy source once glycogen stores are depleted. Recent evidence suggests that ketone bodies may act as endogenous signaling molecules capable of influencing sympathetic activity by acting as meditators of cardiovascular function. In mice, ketones have been reported to suppress sympathetic activity and reduce heart rate (43). In humans, acute ketone body infusion increases myocardial blood flow in healthy humans (ketone concentration ∼3.78 mmol/L; 44) and increases stroke volume and cardiac output in patients with chronic heart failure (ketone concentration ∼3.3 mmol/L; 45). The ketone concentrations reached in the infusion studies can be obtained after an ∼72-h fast (∼3 mmol/L; 41). However, in both of the infusion studies and the 72-h fast, the hyperketonemia was associated with an unexplained increase in heart rate. In our study, we measured a mild but significant increase in ketone bodies (β-OHB ∼0.54 mmol/L) after a 24-h fast, which was associated with lower heart rate, enhanced HRV, and increased estimated stroke volume. Mild increases in ketone bodies may represent an optimal level of ketosis that enhances vagal modulation of the heart. Future studies should further investigate the role of ketones on autonomic cardiovascular control in humans with an emphasis on establishing an exposure-response relationship between ketone concentrations and parasympathetic tone.

### Perspectives and Significance

Intermittent fasting is currently the most popular dieting strategy in the United States. Our current findings provide new mechanistic and potentially prepotent insight into how acute, short-term fasting reduces arterial pressure. An acute 24-h fast decreases overall 24-h blood pressure and heart rate, increases vagal modulation of the heart, and enhances cardiovagal baroreflex sensitivity without altering directly measured peripheral sympathetic nerve activity compared with a period of normal eating. Potential cardiovascular health benefits of fasting are underscored by increased heart rate variability and cardiovagal baroreflex sensitivity both at rest and during the hypertensive phase IV of the Valsalva maneuver.

### Limitations

This study is not presented without limitations. Although we included a population of 25 participants, we report MSNA data from only 12 participants. The microneurography procedure is delicate, and it is sometimes difficult to obtain adequate nerve recordings from some participants. This problem is made worse by the requirement of obtaining adequate nerve recordings from the same person twice. We cannot speculate how inclusion of MSNA for all 25 participants might have impacted our results and interpretation. Initial ABPM fitting and initiation were performed by experimenters, and participants were informed about the specific monitoring process. However, participants were given the opportunity to remove the ABPM for showering, potentially introducing an error upon cuff replacement.

## GRANTS

This work was supported by a grant from the Michigan Space Grant Consortium (to J. E. Gonzalez), an endowment from the Portage Health Foundation (Houghton, MI; to W. H. Cooke), and by an Oregon Health and Science University Fellowship for Diversity in Research (to J. E. Gonzalez).

## DISCLOSURES

No conflicts of interest, financial or otherwise, are declared by the authors.

## AUTHOR CONTRIBUTIONS

J.E.G. and W.H.C. conceived and designed research; performed experiments; analyzed data; interpreted results of experiments; prepared figures; drafted manuscript; edited and revised manuscript; approved final version of manuscript.
